# Measurement of quality of stroke care with national electronic health records: a prospective cohort study during and after the COVID-19 pandemic

**DOI:** 10.1136/bmjopen-2025-114881

**Published:** 2026-05-19

**Authors:** James Farrell, John Nolan, Roger Lambert, Ana Torralbo, Steffen E Petersen, Mevhibe Hocaoglu, Chris Tomlinson, Reecha Sofat, Qi Huang, Evan Kontopantelis, Martin James, Sarah Lessels, Jacqueline A L MacArthur, Angela M Wood, William N Whiteley, Spiros Denaxas, on behalf of the CVD-COVID-UK/COVID-IMPACT Consortium

**Affiliations:** 1 British Heart Foundation Data Science Centre, Health Data Research UK, London, UK; 2 University College London Institute of Health Informatics, London, England, UK; 3 William Harvey Research Institute, Queen Mary University of London, London, England, UK; 4 Cicely Saunders Institute, King’s College London, London, England, UK; 5 University of Liverpool Department of Pharmacology and Therapeutics, Liverpool, England, UK; 6 Clinical Operational Research Unit, University College London, London, UK; 7 The University of Manchester Division of Informatics Imaging and Data Sciences, Manchester, England, UK; 8 University of Exeter Medical School, Exeter, England, UK; 9 Sentinel Stroke National Audit Programme, King’s College London, London, England, UK; 10 Cardiovascular Epidemiology Unit, Department of Public Health and Primary Care, Cambridge, UK; 11 Centre for Clinical Brain Sciences, The University of Edinburgh, Edinburgh, Scotland, UK; 12 Institute of Health Informatics, University College London, London, England, UK; 13 Interdisciplinary Transformation University (ITU), Linz, Austria; 14 National and Kapodistrian University of Athens, Athens, Attica, Greece; 15 Data Science Centre, British Heart Foundation, London, UK

**Keywords:** Stroke, Quality in health care, COVID-19

## Abstract

**Abstract:**

**Objectives:**

To evaluate the value of linked electronic health records (EHRs) for measuring stroke care quality in England before and after the COVID-19 pandemic, focusing on metrics not routinely captured: stroke incidence, dispensing of secondary prevention medications and a proxy of disability—time spent at home after stroke (‘home-time’).

**Design:**

Prospective cohort study using national linked datasets.

**Setting:**

England-wide health data linkage including the Sentinel Stroke National Audit Programme (SSNAP), primary and secondary care, dispensed medications and mortality records, accessed via National Health Service (NHS) England’s Secure Data Environment.

**Participants:**

425 675 adults with a first stroke between 1 January 2020 and 31 December 2023; data were available for 304 210 in primary care, 279 825 in hospital admissions, 220 470 in SSNAP and 59 465 in death records.

**Main outcome measures:**

Annual stroke incidence; first-year medication dispensing rates for antiplatelets, anticoagulants, antihypertensives and lipid-lowering agents (with a 1-month washout period) and home-time at 180 days post stroke.

**Results:**

Stroke ascertainment was highest when combining all sources, with 10.8% of non-fatal ischaemic strokes recorded exclusively in primary care and 19.4% of fatal strokes identified solely through death records. Standardised annual stroke incidence rose from 227.6 (95% CI 226.1 to 229.0) to 244.8 (95% CI 243.4 to 246.3) per 100 000 over the study period including the COVID-19 pandemic. During the COVID-19 lockdown, non-fatal stroke recordings decreased while stroke-related deaths rose, indicating that recording quality was sensitive to shifts in healthcare-seeking behaviour during the pandemic. Among people with ischaemic stroke, 89.1% received an antiplatelet or anticoagulant, 44.5% an antihypertensive and 80.5% a lipid-lowering therapy. For haemorrhagic stroke, these proportions were: for anticoagulants 13.5%, antiplatelets 13.2%, antihypertensives 46.6% and lipid lowering 41.1%. Medication dispensing for stroke prevention declined with increasing age and comorbidity, but varied little by ethnicity, region or pandemic period. Mean home-time within 180 days of stroke was 166.6 (95% CI 166.4 to 166) days, decreasing with greater age (141.4 days for 90 years or older (95% CI 140.7 to 142.1)), deprivation (166.4 days (95% CI 166.1 to 166.6) for most deprived quintile) and stroke severity (137.4 days for National Institutes of Health Stroke Scale (NIHSS) score on arrival over 22 (95% CI 135.8 to 139.1)) and increasing with years from the COVID-19 pandemic 2023 (169.3 days (95% CI 169.0 to 169.5) vs 2020 164.4 days (95% CI 164.1 to 164.7)).

**Conclusions:**

Standardised stroke incidence increased significantly over the study period, highlighting a growing public health burden that persisted despite disruptions due to the pandemic although variation in case ascertainment and stroke coding practices was observed. While secondary prevention coverage for antiplatelets and lipids was high, lower rates of dispensing of antihypertensives, particularly in older and comorbid populations, potentially signal a target for improvement. Home-time represents a sensitive, person-centred outcome that exposes disparities linked to socioeconomic deprivation and clinical severity that can be used to enhance routine stroke audits. These findings justify the expansion of linked EHR infrastructure and the modernisation of governance frameworks to enable the longitudinal evaluation of care quality beyond the COVID-19 era.

**STRENGTHS AND LIMITATIONS OF THIS STUDY**

- This study uses a whole-population linkage of national audit (Sentinel Stroke National Audit Programme), primary care (General Practice Extraction Service Data for Pandemic Planning and Research), hospital (Hospital Episode Statistics) and mortality (Office for National Statistics) records, which maximises case ascertainment compared with routine audits.
- The use of NHS Business Services Authority medication dispensing data potentially provides a more accurate proxy for medication adherence than primary care prescribing records alone.
- The inclusion of primary care records enables capture of patients not admitted to hospital or managed in ambulatory settings, while linkage with hospital records allows longitudinal measurement of ‘home-time’ following stroke.
- Potential misclassification of stroke subtypes and first-ever versus recurrent stroke events remains a risk due to the varying positive predictive value of International Classification of Disease 10 (ICD 10) and Systematized Nomenclature of Medicine Clinical Terms (SNOMED CT) Iconcepts in electronic health records from different healthcare settings.
- The analysis is unable to account for medications prescribed in the private sector or clinical factors not captured in routine electronic health records, such as specific reasons for contraindications, prior tolerance or eligibility to secondary prevention.

## Introduction

 Near-real-time linkage of routine health and medication dispensing records could enhance national stroke audits by improving case ascertainment and reducing the time and cost of data collection. Furthermore, integrating this data allows for the identification of care inequalities and the development of new quality measures, such as 6-month disability indicators and secondary prevention coverage. Here, we test the benefits of linking the Sentinel Stroke National Audit Programme (SSNAP) in England to wider health-systems data during the COVID-19 pandemic, when quality of care was expected to vary substantially.^1^

UK national audits of stroke care like SSNAP measure quality of care for people admitted to hospitals with stroke. In addition, audits provide high-fidelity data on particular diseases that are otherwise not available in UK hospitalisation or death records, hence are particularly useful for public health and epidemiological stroke research. SSNAP makes considerable effort to ensure complete and accurate ascertainment of people admitted to hospitals. This is resource intensive, but despite these efforts, audits may not ascertain everybody affected—for example, the Acute Myocardial Ischaemia National Audit Project did not record myocardial infarction cases primarily ascertained in primary care (eg, from emergency department discharges) and at death.^2^ It is possible that stroke audits also miss some people affected by stroke—for example, those with milder stroke who are not admitted to hospital, people who are looked after outside of a stroke service or those who die very quickly in emergency departments—and may be particularly affected by health emergencies such as the COVID-19 pandemic.

Here, we used linked national electronic health records—including SSNAP, primary care, hospital admissions, dispensed medications and mortality data—to evaluate how stroke ascertainment varied across sources, to assess the impact of the COVID-19 pandemic on stroke recording and care, and to test whether routinely collected data can support quality measurement across three domains: completeness of case capture across datasets to inform future surveillance approaches; implementation of secondary prevention with medication dispensing, including antiplatelets, antihypertensives and statins; use of ‘home-time’ in the first 6 months post stroke as a proxy for functional outcome.

## Methods

### Study design and data sources

#### Study population

We implemented a prospective cohort study and identified people over 18 years with a diagnosis of stroke between 1 January 2020 and 31 December 2023 and who were registered with a general practitioner, living in England, had at least one record in General Practice Extraction Service Data for Pandemic Planning and Research (GDPPR) and had a valid pseudoidentifier.^3^ Residents of England were identified with the lower layer super output area (LSOA) of residence.

We linked each person to Hospital Episode Statistics (HES), GDPPR, medication dispensing in primary care (NHS Business Services Authority (NHSBSA)),^4^ and Office for National Statistics death registrations (ONS Deaths).^5^ Data were accessed via the BHF Data Science Centre’s CVD-COVID-UK/COVID-IMPACT Consortium in NHS England’s Secure Data Environment (SDE) service for England.^3^ Individual data sources were linked by NHS Digital using the Master Person Service in combination with the Personal Demographics Service and stringent data quality controls were applied upstream by NHS Digital to ensure data linkages were accurate.

#### Identifying strokes

The first stroke event (index stroke) was defined as the earliest record across multiple sources: hospital admissions in SSNAP or HES-Admitted Patient Care (APC), primary care records in GDPPR or the date of death in ONS Deaths. Stroke records from different sources were considered the same event if their dates fell within 30 days of the index stroke date, to allow for minor discrepancies in recording. Individuals with any stroke records in these sources prior to the study start date were excluded from the analyses.

We classified index strokes into three mutually exclusive phenotypes: ischaemic stroke, haemorrhagic stroke and unknown stroke type. Stroke type was determined using different data sources. In SSNAP, stroke type was recorded in the field ‘S2 stroke type’. In HES-APC and ONS Deaths, stroke was identified using ICD-10 codes, I63 for ischaemic stroke (cerebral infarction), I61 for haemorrhagic stroke (intracerebral haemorrhage) and I64 for unknown stroke type (not specified as haemorrhage or infarction). In HES-APC, we used the ICD-10 from the primary diagnosis of any episode within a spell and, in ONS Deaths, from the cause of death. In GDPPR, stroke was identified and classified with SNOMED-CT concepts as outlined in the supplement. To consolidate stroke type across sources, we prioritised records in the following order: SSNAP, HES-APC, GDPPR and ONS Deaths. If a source with the highest priority contained both ischaemic and haemorrhagic stroke records, the stroke type was classified as unknown. Index strokes were further classified as fatal (death from any cause within 30 days) or non-fatal.

#### Covariate definitions

Age, sex and ethnicity were obtained from the most recent non-missing value across primary care (GDPPR) and secondary care (HES-APC). When values conflicted between sources, priority was given to primary care. Ethnicity was classified in the ONS categories: white, Asian or Asian British, black or black British, mixed or other.^6^

Socioeconomic deprivation was derived by linking the 2011 LSOAs to the 2019 Index of Multiple Deprivation and stratified into quintiles from most to least deprived.

GDPPR and HES-APC (primary and non-primary diagnoses) were used to define prevalent conditions between birth and a day prior to the earliest stroke: atrial fibrillation (AF), cancer, dementia, type 2 diabetes, hypertension, obesity, chronic obstructive pulmonary disease, stable angina, depression, deep vein thrombosis and chronic kidney disease (CKD). Hypercholesterolaemia was defined with GDPPR alone. COVID-19 infection was defined with HES-APC, GDPPR and COVID-19 testing data (Second Generation Surveillance System and the UK Non-hospital COVID-19 testing dataset (also known as Pillar 2)).^7^

We defined smoking status, body mass index, systolic blood pressure (SBP), estimated glomerular filtration rate (eGFR), glycated haemoglobin, total cholesterol and high density lipoprotein cholesterol by extracting SNOMED concepts using previously validated phenotyping^7^ algorithms from GDPPR between 2 years and 1 day prior to the earliest recorded stroke and within predefined value ranges (supplied in online supplemental appendix).

#### Outcome definitions

Home-time (or time out of hospital) was defined as days alive and not in hospital in the 180 days from first stroke record date, where hospital time was defined as the sum of the duration of all admission to discharge spells (or end of follow-up) within the first 6 months. We defined mortality as death of any cause within 30 days of the first recorded stroke.

We extracted relevant dispensed medications (eg, statins, antihypertensives, anticoagulants and antiplatelets) from NHSBSA using previously validated phenotyping algorithms.^8^ Previous medications were defined as prescriptions in the year prior to the earliest stroke event. Incident prescriptions were defined as a prescription from 1 month after discharge date (for hospitalised stroke) or earliest stroke date (if not hospitalised) from 1 month to 1 year of the index event.

Phenotyping algorithms, codelists and related metadata are provided in the Supplementary Materials (online supplemental table S6).

### Statistical analyses

We used descriptive statistics to summarise patient populations and characteristics in stroke that were common or unique to each data source. For the earliest stroke, we plot and report the differences between event dates recorded in HES-APC, GDPPR and SSNAP.

We used UpSet plots to describe the agreement between initial stroke records in GDPPR, HES-APC, SSNAP and ONS Deaths. Crude and adjusted mean home-time was calculated for the overall cohort and stratified by stroke type, demographics, comorbidities, data source and for patients in SSNAP, NIHSS on arrival and modified Rankin score at discharge. Both unadjusted and adjusted estimates were reported with a multivariable linear model.

The 1-year cumulative incidence of poststroke dispensed medications for anticoagulants, antiplatelets, antihypertensives and lipid-lowering drugs was calculated, with death as a competing event. Monthly stroke incidence rates between 2020 and 2023 were age and sex standardised to the European Standard Population (ESP) using the direct method. Cumulative incidence was stratified by demographic factors, Charlson comorbidity index, data source, AF and prestroke medication use.

A multivariable cause-specific Cox proportional hazards model was used to estimate HRs for covariates and to calculate the covariate-adjusted cumulative incidence of the first poststroke dispensed medication. Formal evaluation of the proportional hazards assumption was not performed, as the estimated HRs were intended to summarise time-averaged effects over the follow-up period rather than to explore time-varying associations. We conducted complete data analysis.

### Data access and tools

The data used in this study are available in NHS England’s Secure Data Environment (SDE) service for England, but as restrictions apply they are not publicly available (https://digital.nhs.uk/services/secure-data-environment-service). The CVD-COVID-UK/COVID-IMPACT programme, led by the BHF Data Science Centre (https://bhfdatasciencecentre.org/), received approval to access data in NHS England’s SDE service for England from the Independent Group Advising on the Release of Data (https://digital.nhs.uk/about-nhs-digital/corporate-information-and-documents/independent-group-advising-on-the-release-of-data) via an application made in the Data Access Request Service (DARS) Online system (ref. DARS-NIC-381078-Y9C5K) (https://digital.nhs.uk/services/data-access-request-service-dars/dars-products-and-services). The CVD-COVID-UK/COVID-IMPACT Approvals and Oversight Board (https://bhfdatasciencecentre.org/areas/cvd-covid-uk-covid-impact/) subsequently granted approval to this project to access the data within NHS England’s SDE service for England. The deidentified data used in this study were made available to accredited researchers only. Those wishing to gain access to the data should contact bhfdsc@hdruk.ac.uk in the first instance. The plan, phenotype definitions and code for this analysis are published on GitHub (https://github.com/BHFDSC/CCU005_08).

### Role of the funding source

The funders had no role in study design, data collection, data analysis, data interpretation of data or writing of the report.

### Patient and public involvement

A panel of six patient and public representatives directly affected by stroke or cardiovascular disease provided perspective and input on this research proposal, plain English summary and research outputs, with RL contributing as coauthor of this manuscript.

## Results

Between 1 January 2020 and 31 December 2023, we identified 425 675 people with a first stroke of whom 304 210 (71.5%) had a record in primary care, 279 825 (65.7%) in hospital admissions, 220 470 (51.8%) in SSNAP and 59 465 (14.0%) in ONS death records, with median ages of 74.0 years, 76.0 years, 76.0 years and 83.0 years, respectively. A small majority of strokes in GDPPR, HES, SSNAP were in men (53.5%, 52.5%, 52.9%, respectively) and in death records, a small majority were in women (55.4%). Distribution of ethnicity, region and deprivation were similar between sources. Where the stroke was identified in death records, patients tended to be less obese, with worse eGFR, lower cholesterol and SBP, with a greater proportion affected by comorbidities (particularly AF, CKD, cancer and dementia) than in other sources (table 1).

**Table 1:** Patient characteristics, demographics and stroke-related data for individuals with a first stroke between 2020 and 2023, stratified by the presence of a stroke record in the General Practice Extraction Service Data for Pandemic Preparedness (GDPPR), Hospital Episode Statistics Admitted Patient Care (HES-APC), Sentinel Stroke National Audit Programme (SSNAP) or Office for National Statistics (ONS) Deaths

		GDPPR	HES-APC	SSNAP	ONS Deaths	All sources
Individuals, n (% all sources)		304 210 (71.5)	279 825 (65.7)	220 470 (51.8)	59 465 (14.0)	425 675 (100.0)
Stroke type	Ischaemic	216 295 (71.1)	237 600 (84.9)	193 680 (87.8)	28 990 (48.8)	296 730 (69.7)
	Haemorrhagic	30 070 (9.9)	38 760 (13.9)	26 700 (12.1)	15 270 (25.7)	55 490 (13.0)
	Unknown	57 845 (19.0)	3460 (1.2)	90 (0.0)	15 205 (25.6)	73 455 (17.3)
Age (years)	Median (IQR)	74.0 (62.3–82.6)	76.0 (64.6–84.4)	76.0 (64.8–84.3)	83.0 (74.2–89.5)	75.5 (63.8–84.1)
Sex	Male	162 830 (53.5)	146 890 (52.5)	116 720 (52.9)	26 500 (44.6)	222 245 (52.2)
Ethnicity	White	272 065 (89.4)	251 220 (89.8)	198 745 (90.1)	54 595 (91.8)	380 625 (89.4)
	Asian	16 885 (5.6)	14 895 (5.3)	11 495 (5.2)	2390 (4.0)	23 380 (5.5)
	Black	8715 (2.9)	7990 (2.9)	6000 (2.7)	1180 (2.0)	12 375 (2.9)
	Mixed	2425 (0.8)	2080 (0.7)	1540 (0.7)	335 (0.6)	3325 (0.8)
	Other	3150 (1.0)	2610 (0.9)	1920 (0.9)	435 (0.7)	4310 (1.0)
	Unknown	970 (0.3)	1030 (0.4)	775 (0.4)	535 (0.9)	1660 (0.4)
Index of Multiple Deprivation 2019 quintiles	1 (most deprived)	58 875 (19.4)	56 045 (20.0)	44 100 (20.0)	11 390 (19.2)	84 165 (19.8)
	2	59 640 (19.6)	55 405 (19.8)	43 405 (19.7)	11 580 (19.5)	84 125 (19.8)
	3	62 700 (20.6)	57 305 (20.5)	45 050 (20.4)	12 480 (21.0)	87 370 (20.5)
	4	62 920 (20.7)	57 145 (20.4)	45 130 (20.5)	12 345 (20.8)	87 210 (20.5)
	5 (least deprived)	60 070 (19.7)	53 925 (19.3)	42 785 (19.4)	11 670 (19.6)	82 805 (19.5)
Smoking status	Current smoker	40 105 (13.2)	34 845 (12.5)	27 510 (12.5)	5445 (9.2)	53 210 (12.5)
	Ex-smoker	76 770 (25.2)	70 305 (25.1)	55 705 (25.3)	16 030 (27.0)	107 990 (25.4)
	Never smoked	92 825 (30.5)	83 375 (29.8)	65 470 (29.7)	18 695 (31.4)	129 430 (30.4)
	Unknown	94 515 (31.1)	91 305 (32.6)	71 790 (32.6)	19 290 (32.4)	135 040 (31.7)
Body mass index	Median (IQR)	27.3 (23.9–31.2)	27.1 (23.7–31.2)	27.2 (23.8–31.2)	25.1 (21.6–29.2)	27.0 (23.6–31.0)
eGFR	Median (IQR)	70.0 (56.0–83.0)	67.0 (53.0–82.0)	67.0 (53.0–81.0)	62.0 (47.0–78.0)	68.0 (54.0–82.0)
Glycated haemoglobin	Median (IQR)	41.0 (37.0–48.0)	41.0 (38.0–49.0)	41.0 (38.0–48.0)	41.0 (37.0–47.0)	41.0 (37.0–48.0)
Systolic blood pressure	Median (IQR)	136.0 (126.0–148.0)	137.0 (126.0–148.0)	137.0 (126.0–148.0)	133.0 (120.0–145.0)	136.0 (125.0–147.0)
HDL cholesterol	Median (IQR)	1.3 (1.1–1.6)	1.3 (1.1–1.6)	1.3 (1.1–1.6)	1.3 (1.1–1.7)	1.3 (1.1–1.6)
Total cholesterol	Median (IQR)	3.4 (2.7–4.2)	3.3 (2.7–4.2)	3.3 (2.7–4.2)	3.1 (2.5–3.9)	3.3 (2.7–4.2)
Angina	Yes	48 370 (15.9)	48 665 (17.4)	38 340 (17.4)	12 970 (21.8)	72 480 (17.0)
Arrhythmia	Yes	4465 (1.5)	5000 (1.8)	3395 (1.5)	2545 (4.3)	8150 (1.9)
Atrial fibrillation or myocardial infarction	Yes	90 040 (29.6)	99 525 (35.6)	77 400 (35.1)	29 785 (50.1)	141 030 (33.1)
Diabetes	Yes	88 280 (29.0)	86 050 (30.8)	66 960 (30.4)	19 050 (32.0)	127 320 (29.9)
Hypercholesterolaemia	Yes	25 965 (8.5)	24 800 (8.9)	19 550 (8.9)	5580 (9.4)	37 515 (8.8)
Hypertension	Yes	207 235 (68.1)	203 950 (72.9)	159 755 (72.5)	46 645 (78.4)	299 035 (70.2)
Obesity	Yes	61 395 (20.2)	58 675 (21.0)	45 415 (20.6)	11 150 (18.7)	86 280 (20.3)
Cancer	Yes	72 570 (23.9)	69 470 (24.8)	53 720 (24.4)	18 980 (31.9)	106 920 (25.1)
Chronic kidney disease	Yes	87 555 (28.8)	93 005 (33.2)	70 600 (32.0)	30 465 (51.2)	139 235 (32.7)
Liver disease	Yes	4405 (1.4)	4230 (1.5)	3085 (1.4)	1360 (2.3)	6910 (1.6)
Chronic obstructive pulmonary disease	Yes	39 305 (12.9)	39 490 (14.1)	30 650 (13.9)	11 135 (18.7)	59 630 (14.0)
Dementia	Yes	22 675 (7.5)	25 310 (9.0)	18 875 (8.6)	13 765 (23.1)	41 330 (9.7)
Depression	Yes	83 235 (27.4)	73 255 (26.2)	57 025 (25.9)	15 575 (26.2)	115 745 (27.2)
Deep vein thrombosis	Yes	7640 (2.5)	7965 (2.8)	5835 (2.6)	2720 (4.6)	12 525 (2.9)
Charlson Comorbidity Index	0	60 850 (20.0)	23 700 (8.5)	24 310 (11.0)	4610 (7.8)	70 635 (16.6)
	1–2	115 525 (38.0)	106 585 (38.1)	83 595 (37.9)	15 690 (26.4)	154 310 (36.3)
	3–4	73 680 (24.2)	81 880 (29.3)	62 610 (28.4)	16 720 (28.1)	108 580 (25.5)
	5+	54 155 (17.8)	67 655 (24.2)	49 955 (22.7)	22 445 (37.7)	92 150 (21.6)
COVID-19 within 14 days prior to stroke	Yes	8230 (2.7)	10 110 (3.6)	6475 (2.9)	3825 (6.4)	14 965 (3.5)
History of COVID-19	Yes	45 765 (15.0)	42 980 (15.4)	31 445 (14.3)	12 625 (21.2)	69 150 (16.2)
Pre-stroke anticoagulants	Yes	45 645 (15.0)	48 925 (17.5)	38 260 (17.4)	16 140 (27.1)	72 605 (17.1)
Pre-stroke antihypertensives	Yes	121 915 (40.1)	115 805 (41.4)	91 845 (41.7)	24 240 (40.8)	172 175 (40.4)
Pre-stroke antiplatelets	Yes	103 560 (34.0)	82 200 (29.4)	66 300 (30.1)	16 465 (27.7)	138 315 (32.5)
Pre-stroke lipid lowering drugs	Yes	155 180 (51.0)	136 375 (48.7)	109 190 (49.5)	26 785 (45.0)	212 980 (50.0)
Length of stay (days)	Median (IQR)	4.0 (2.0 to 12.0)	5.0 (2.0 to 16.0)	5.0 (2.0 to 14.0)	6.0 (2.0 to 12.0)	5.0 (2.0 to 15.0)
NIHSS score on arrival	0–4	89 305 (29.4)	106 580 (38.1)	116 295 (52.7)	3575 (6.0)	116 295 (27.3)
	5–10	38 520 (12.7)	49 950 (17.9)	53 335 (24.2)	3580 (6.0)	53 335 (12.5)
	11–15	11 510 (3.8)	17 230 (6.2)	18 370 (8.3)	3155 (5.3)	18 370 (4.3)
	16–21	9670 (3.2)	16 680 (6.0)	17 655 (8.0)	5510 (9.3)	17 655 (4.1)
	22+	6120 (2.0)	13 615 (4.9)	14 680 (6.7)	7650 (12.9)	14 680 (3.4)
	(missing)	149 085 (49.0)	75 770 (27.1)	140 (0.1)	35 990 (60.5)	205 345 (48.2)
Rankin score at discharge	0	15 660 (5.1)	17 800 (6.4)	19 755 (9.0)	135 (0.2)	19 755 (4.6)
	1	25 930 (8.5)	29 555 (10.6)	32 055 (14.5)	135 (0.2)	32 055 (7.5)
	2	20 820 (6.8)	23 510 (8.4)	25 335 (11.5)	175 (0.3)	25 335 (6.0)
	3	16 440 (5.4)	19 375 (6.9)	20 690 (9.4)	325 (0.5)	20 690 (4.9)
	4	12 865 (4.2)	16 635 (5.9)	17 715 (8.0)	740 (1.2)	17 715 (4.2)
	5	5720 (1.9)	8350 (3.0)	8895 (4.0)	2055 (3.5)	8895 (2.1)
	6	3960 (1.3)	18 015 (6.4)	20 160 (9.1)	17 145 (28.8)	20 160 (4.7)
	(missing)	202 815 (66.7)	146 580 (52.4)	75 870 (34.4)	38 765 (65.2)	281 070 (66.0)

The denominator for the top row is the total number of individuals across all sources (n=4 25 675); for subsequent rows, denominators are the number of individuals recorded in each data source (304 210 in GDPPR, 279 825 in HES-APC, 220 470 in SSNAP and 59 465 in ONS).

eGFR, estimated glomerular filtration rate; HDL, high density lipoprotein.

The estimated incidence of stroke during the period from 1 January 2020 to 31 December 2023 was highest for a comprehensive ascertainment of stroke from all sources, compared with individual sources alone. The incidence rate per 100 000 person years, age-standardised and sex-standardised to the ESP, for strokes identified from all four sources was 227.6 (226.1, 229.0) in 2020 and rose to 244.8 (243.4, 246.3) in 2023 (online supplemental table S1).

The percentage of people with ischaemic, haemorrhagic and unknown stroke types was, in primary care, 71.1%, 9.9% and 19.0%; in hospital statistics, 84.9%, 13.9% and 1.2%; in SSNAP, 87.8%, 12.1% and <0.1%; and in death records, 48.8%, 25.7% and 25.6%, respectively. Using the harmonised stroke type, these proportions were 69.7%, 13.0% and 17.3%. (table 1)

**Figure 1:**
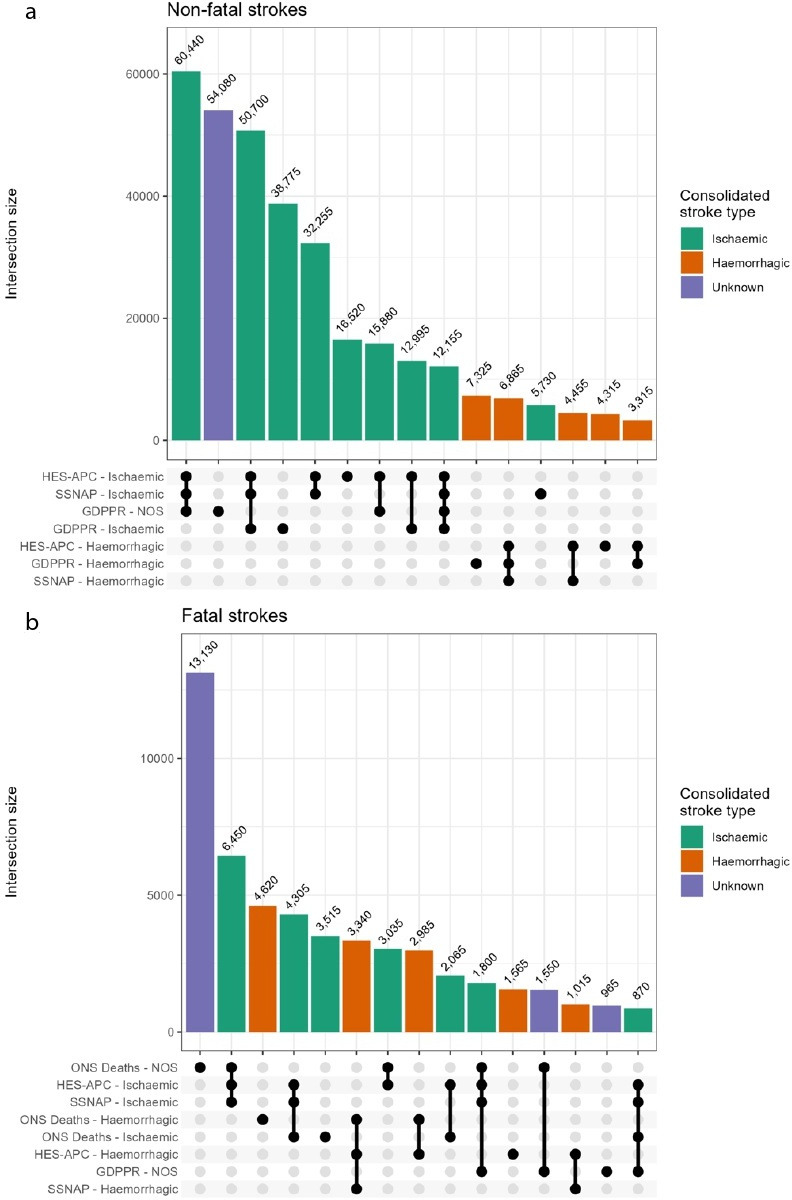
(**a**) UpSet plot of the 15 most frequent intersections of stroke events across GDPPR, HES-APC, SSNAP and (b) ONS Deaths by stroke type. Counts represent individuals with non-fatal (n=3 58 025) and fatal strokes (n=67 650). GDPPR, General Practice Extraction Service Data for Pandemic Preparedness; HES-APC, Hospital Episode Statistics Admitted Patient Care; NOS, not otherwise specified; ONS, Office for National Statistics; SSNAP, Sentinel Stroke National Audit Programme.

Non-fatal ischaemic strokes in SSNAP were almost always recorded in HES-APC as ischaemic or unknown and were rarely recorded without at least one other corroborating source (3.6% of non-fatal strokes). Haemorrhagic strokes in SSNAP were very rarely recorded without another corroborating source (0.6% of non-fatal strokes) and were almost always recorded as haemorrhagic in primary or hospital records. There were a significant number of patients with a diagnosis of ischaemic stroke who were only recorded in primary care (10.8% of non-fatal strokes) or hospital records (4.6% of non-fatal strokes), and the largest number of haemorrhagic strokes were only recorded in primary care (2.0% of non-fatal strokes) (figure 1). The largest number of fatal strokes (19.4%) had no subtype information and were only recorded in death records. Death records were the only source of subtype information for 4620 haemorrhagic strokes (6.8% of fatal strokes) and 3515 ischaemic strokes (5.2% of fatal strokes) (onlinesupplemental tables S2a,b  S3a,b), (figure 1, online supplemental figure S1).

The median difference in stroke onset date between sources, compared from the earliest recorded date within each source, was modest. Between primary care and hospital episodes it was 0 days (IQR 0–4), primary care and SSNAP 0 days (IQR 0–4), SSNAP and hospital episodes 0 days (IQR 0–0) (online supplemental figure S2).

**Figure 2:**
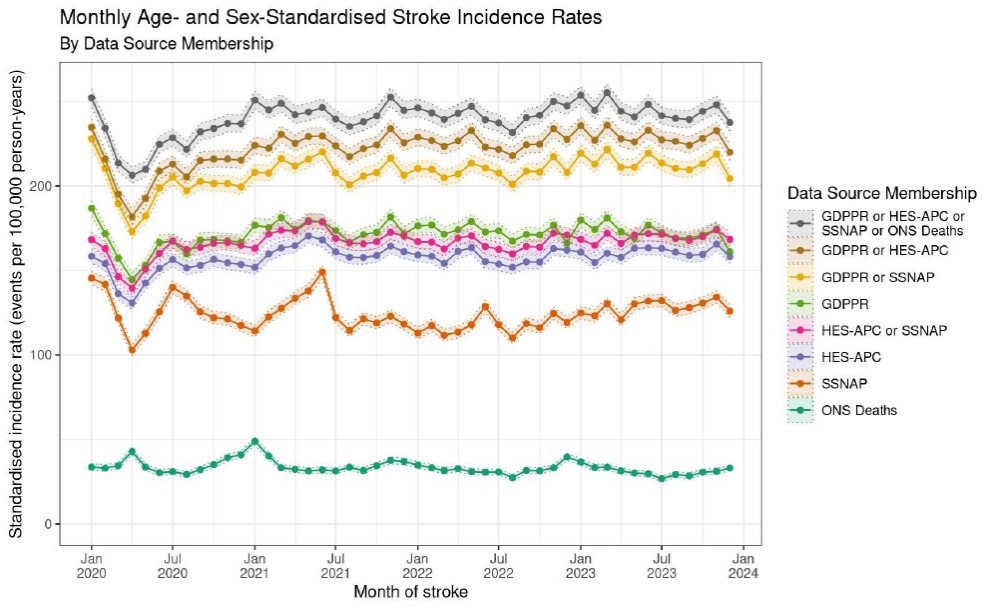
Monthly age-standardised and sex-standardised incidence rates of stroke (standardised to the European Standard Population) between 2020 and 2023 for individuals aged 18 years and older by data source. COVID-19 lockdowns were March 2020–July 2020, and November 2020–December 2020. GDPPR, General Practice Extraction Service Data for Pandemic Preparedness; HES-APC, Hospital Episode Statistics Admitted Patient Care; ONS, Office for National Statistics; SSNAP, Sentinel Stroke National Audit Programme.

The highest incidence of stroke in an individual source for calendar year 2023 was recorded in primary care at 172.6 (171.3, 173.8) per 100 000, followed by hospital episodes (160.5 (159.4, 161.7)), SSNAP (128.2 (127.2, 129.3)), then death records (31.1 (30.6, 31.7)). During early 2020 (the lockdown period of the COVID-19 pandemic), non-fatal strokes recorded during healthcare encounters fell, although deaths due to stroke rose. During the pandemic, incidence of stroke classified as ischaemic fell by more than strokes classified as unknown or haemorrhagic (figure 2).

**Figure 3:**
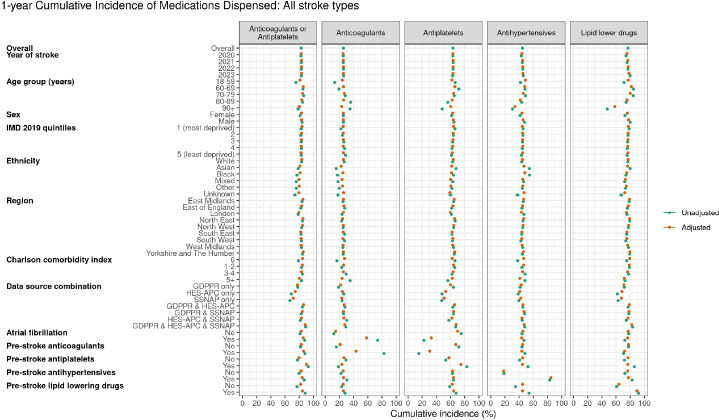
Unadjusted and covariate-adjusted 1-year cumulative incidence of anticoagulants, antiplatelets, antihypertensives and lipid-lowering drugs dispensed to individuals with non-fatal strokes identified through GDPPR, HES-APC or SSNAP (n=3 48 300). GDPPR, General Practice Extraction Service Data for Pandemic Preparedness; HES-APC, Hospital Episode Statistics Admitted Patient Care; SSNAP, Sentinel Stroke National Audit Programme.

For ischaemic stroke, within the first year of stroke, the proportion of people dispensed an antiplatelet or anticoagulant was 89.1%, anticoagulant medication was 27.0%, antiplatelet medication was 68.9%, antihypertensive medication 44.5% and lipid-lowering medications 80.5% (figure 3, online supplemental figure S3a). For haemorrhagic stroke, the proportion of people dispensed a prescription of anticoagulant medication within the first year was 13.5%, antiplatelet medication was 13.2%, antihypertensive medication 46.6% and lipid-lowering medications 41.1% (figure 3, online supplemental figure S3b).

After adjusting for other stroke characteristics, older people were less likely to be dispensed lipid-lowering or antihypertensive medication but not anticoagulants or antiplatelets, with no large differences by ethnicity or consistent differences for different drug classes by region. People with the greatest number of comorbidities were less likely to be dispensed any of the medicine classes compared with people with fewer comorbidities, although the absolute differences were small. 77.1% of people with prior AF and ischaemic stroke were dispensed an anticoagulant in the following year. For years 2020–2023, the 1-year cumulative incidence of both anticoagulants and antiplatelets dispensed was similar at 25% and 63%, respectively, while antihypertensives increased from 43.1% to 46.0% and lipid-lowering drugs from 74.1% to 79.9% (online supplemental table 4a–c). There was no important difference in 2020 compared with 2021–2023.

**Figure 4:**
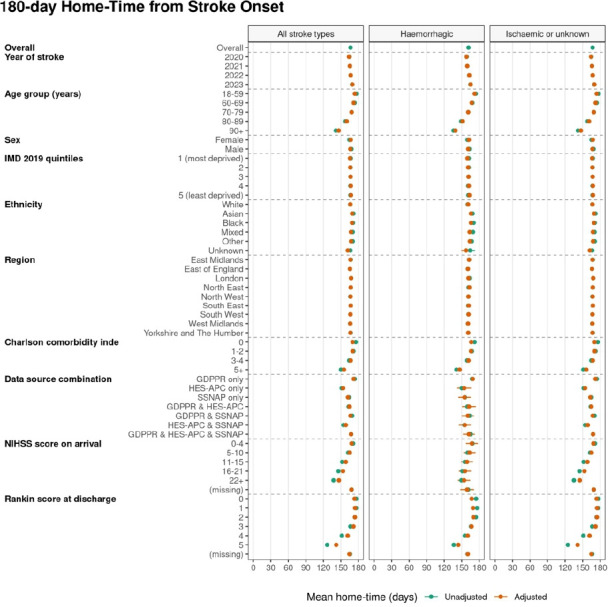
Unadjusted and covariate-adjusted mean 180-day home-time from stroke onset in non-fatal strokes identified through GDPPR, HES-APC or SSNAP, stratified by all stroke types (n=3 53 325), haemorrhagic strokes (n=35 165) and ischaemic or unknown stroke types (n=318. GDPPR, General Practice Extraction Service Data for Pandemic Preparedness; HES-APC, Hospital Episode Statistics Admitted Patient Care; SSNAP, Sentinel Stroke National Audit Programme.

The mean home-time in the first 180 days post stroke was 166.6 days, with good evidence of less home-time with increasing age, deprivation, comorbidity and NIHSS score on arrival and important differences by region in crude and adjusted analysis (online supplemental table S4a–c). Home-time within 180 days post stroke increased with time since the COVID-19 pandemic from 164.4 days (2020) to 165.5 days (2021), 166.8 days (2022) and 169.3 days (2023) (figure 4, online supplemental table S5a)

After adjusting for other characteristics (age, comorbidities and NIHSS), the numbers of days of home-time in 180 days after stroke onset was fewer—compared with patients who were 15–59 years old—for patients aged 60–69 years (by 2.1 days); 70–79 years (by 5.6 days); 80–89 years (by 13.8 days) and 90+ years (by 27.6 days). Compared with women, men had 0.6 fewer days at home; compared with people in the least deprived fifth, those in the most deprived fifth had 1.6 days fewer at home; compared with those with no comorbidities, those with a Charlson score of 5 or more had 14.7 days less at home; and compared with those with an NIHSS score of 0–4 (mild stroke) the number of days fewer at home was, for people with an NIHSS score of 5–10, 3.3 days; 11–15, 9.9 days; 16–21, 14.7 days and >22 was 21.7 days (online supplemental table S5b). Compared with people with a white ethnicity, the numbers of days more at home was, for those: with an Asian ethnicity, 3.0 days; with a black ethnicity 2.8 days; those with a mixed ethnicity 1.1 days, those with other ethnicity 1.2 days and for those with an unknown ethnicity, 4.4 fewer days at home. There were modest differences in home-time between regions, with, compared with the East Midlands, people in the East of England spending 0.9 fewer days at home and people in London spending 0.9 days more at home. Compared with 2020, the number of days at home was 5.8 days more in 2023 (figure 4, online supplemental table S5b).

## Discussion

While each individual data source captured stroke events that were uniquely recorded in that source, the overall concordance of stroke events between sources was very high (median 0 days between records) indicating both a good level of data linkage across sources and quality within sources. We additionally demonstrate, with a comprehensive ascertainment of stroke in all available sources in England, that during the study period (2020–2023) most ischaemic stroke patients were dispensed an anticoagulant or antiplatelet agent. However, we observed lower dispensing of guideline-recommended antihypertensives (40%), statins (80%) and anticoagulants in those with AF (77%), particularly in older people or those with more comorbidities.^9^ There was no major change in the proportion of people treated during the COVID-19 period. Home-time in the first 6 months, an indicator of post-stroke disability, was lower in older people, those with a more severe stroke or more comorbidities, with modest differences by region, sex and ethnicity. After the COVID-19 period, people spent more time at home.

Secondary prevention is key to reducing stroke incidence. While population-wide prescriptions of antihypertensive and lipid-lowering drugs fell during the COVID-19 pandemic,^8^ in stroke patients we found no major difference in dispensed medicines by year or by patient characteristics. This indicates a longer-term and systemic problem not due to the pandemic.^10^ The evidence is clear that targeting a SBP of ≤125 mm Hg^11^ in all patients with stroke (whether ischaemic or haemorrhagic) reduces recurrent stroke and that most stroke patients have a blood pressure over the threshold for hypertension diagnosis at presentation.^12^ It is most likely that antihypertensives have substantially lower rates of dispensing—particularly in older people who stand to gain the most—and that many more patients could benefit. While these findings suggest a potential gap in secondary prevention, the use of dispensing data alone cannot account for clinical contraindications, patient preferences or end-of-life care goals that may appropriately preclude certain medications from being prescribed. Consequently, the observed lower rates of dispensing may reflect, in part, appropriate clinical decision-making that is not captured in routine EHR records.

Linked data for measuring quality of care are essential for a learning health system.^13^ Here, we developed a large, linked dataset with ascertainment of quality beyond the hospital system to primary care and pharmacy. However, this work is unlikely to be replicable routinely beyond the pandemic unless the recommendations of the Sudlow review^14^—to allow national linkage of primary care data—are implemented. No timeline is available for providing access to such data, meaning that patients with stroke who do not become inpatients (eg, discharged from emergency departments or looked after in an ambulatory transient ischaemic attack/stroke clinic or diagnosed in primary care) are missing from national measurement of quality. This is particularly important for pandemic preparedness as our work shows that using a single source for ascertaining stroke, for example, only hospitalisation data, would underestimate the number of people with stroke in England.

Our analysis has a number of strengths. First, it ascertains stroke patients in the whole English population who are registered in primary care (almost the entire population^15^). Second, we ascertained stroke from multiple overlapping sources, including the national quality register of hospitalised stroke, hospital administrative data and primary care. The date of stroke was largely consistent between sources. Third, we used previously developed and validated code lists to identify strokes. Fourth, we were able to ascertain all relevant secondary prevention medicines dispensed to patients, where a submission had been made for reimbursement from NHS England, most prescriptions in the population and a more accurate proxy of compliance than prescribing data.

Our analysis has several weaknesses. First, we were unable to check the coded diagnosis of stroke against written records. The diagnosis of stroke in health systems data in the UK,^16^ compared with manual review of records, has good positive predictive value, when in the primary position in hospital records (94%), is reasonable in primary care (80%), although is less good in death records (57%) and, on average, is less good for haemorrhagic (52%) than ischaemic stroke (83%). This is important, because risk factors and case fatality differ by subtype. Accurate clinical coding is important for all health systems, and methods should be improved with greater use of clinicians or automated coding systems to aid clinical coders.^17^ Second, we did not include recurrent stroke, because differentiating further consultations with the first stroke is difficult to separate from recurrent stroke in records. Third, we examined NHS dispensed rather than prescribed medicines. This will miss medicines that were prescribed but not dispensed, which in some cases is a substantial proportion.^18^ A further limitation is that our data lack granular information on clinical intent, medication intolerance and contraindications. We cannot distinguish between a failure to prescribe and a deliberate clinical decision based on patient frailty or preferences. It will also miss prescriptions from the private sector which are not collected through NHS reimbursement schemes. Fourth, in this manuscript, we did not examine in detail differences by ethnicity or geography and so may have missed communities who were particularly disadvantaged. Fifth, while home-time does correlate with disability and symptoms in this analysis and others, it is influenced by other factors such as family needs and does not directly measure impairment.^19^,^21^ Sixth, our estimates of stroke incidence were high, compared with population-based incidence studies with bespoke measurement of stroke. This may be because there is overuse of stroke codes in primary care data or that differentiating first-ever from recurrent stroke is difficult. This emphasises the need for improved clinical coding throughout healthcare pathways. Seventh, we used data provided by SSNAP under the Notice under Regulation 3 (4) of the Health Service (Control of Patient Information) Regulations 200, that under provisioned records because of truncated monthly uploads to the SDE. Planning mechanisms for complete flow of data would prepare for the next health emergency.

Our analysis has several implications for health policy makers and stroke clinicians. Secondary prevention should be a priority for healthcare quality improvement for patients with stroke and investigation of the clinical, system and patient related factors leading to lower rates of secondary prevention medication should be research priorities. Despite the disruption caused by the COVID-19 pandemic on secondary prevention, we have shown here that potential longer-term gaps in secondary prevention exist that should be addressed. Home-time is an indicator of disability which could be added to the SSNAP and the stroke audit in Scotland for all patients, if it is interpreted carefully. Lastly, the use of linked health data—with an awareness of its limitations—is a promising avenue for audits of care for people with stroke and other conditions, to maximise ascertainment, streamline efficiency of data collection and enhance utility.

## Supplementary material

10.1136/bmjopen-2025-114881 Supplementary file 1

10.1136/bmjopen-2025-114881 Supplementary file 2

## Data Availability

Data may be obtained from a third party and are not publicly available.

## References

[1] Primary S (2014). Sentinel stroke national audit programme (SSNAP). https://www.hqip.org.uk/wp-content/uploads/2019/06/Ref-142-SSNAP-Annual-Report-FINAL.pdf.

[2] Herrett E, Shah AD, Boggon R (2013). Completeness and diagnostic validity of recording acute myocardial infarction events in primary care, hospital care, disease registry, and national mortality records: cohort study. BMJ.

[3] Wood A, Denholm R, Hollings S (2021). Linked electronic health records for research on a nationwide cohort of more than 54 million people in England: data resource. BMJ.

[4] (2025). Dispensing data. https://www.nhsbsa.nhs.uk/prescription-data/dispensing-data.

[5] Campbell A (2020). Quality of mortality data during the coronavirus pandemic, England and wales - Office for National Statistics. https://www.ons.gov.uk/peoplepopulationandcommunity/birthsdeathsandmarriages/deaths/articles/qualityofmortalitydataduringthecoronaviruspandemicenglandandwales/2020.

[6] (2025). List of ethnic groups. https://www.ethnicity-facts-figures.service.gov.uk/style-guide/ethnic-groups/.

[7] Thygesen JH, Tomlinson C, Hollings S (2022). COVID-19 trajectories among 57 million adults in England: a cohort study using electronic health records. Lancet Digit Health.

[8] Dale CE, Takhar R, Carragher R (2023). The impact of the COVID-19 pandemic on cardiovascular disease prevention and management. Nat Med.

[9] (2022). National clinical guideline for stroke.

[10] Whitty CJM, Smith G, McBride M (2023). Restoring and extending secondary prevention. BMJ.

[11] (2023). National clinical guideline for stroke.

[12] Fischer U, Cooney MT, Bull LM (2014). Acute post-stroke blood pressure relative to premorbid levels in intracerebral haemorrhage versus major ischaemic stroke: a population-based study. Lancet Neurol.

[13] Cadilhac DA, Bravata DM, Bettger JP (2023). Stroke Learning Health Systems: A Topical Narrative Review With Case Examples. Stroke.

[14] Sudlow C (2023). The sudlow review. https://www.hdruk.ac.uk/helping-with-health-data/the-sudlow-review/.

[15] (2025). Patients registered at a GP practice. https://digital.nhs.uk/data-and-information/publications/statistical/patients-registered-at-a-gp-practice/february-2025.

[16] Rannikmäe K, Ngoh K, Bush K (2020). Accuracy of identifying incident stroke cases from linked health care data in UK Biobank. Neurology (ECronicon).

[17] Dong H, Falis M, Whiteley W (2022). Automated clinical coding: what, why, and where we are?. *NPJ Digit Med*.

[18] Gardner TL, Dovey SM, Tilyard MW (1996). Differences between prescribed and dispensed medications. N Z Med J.

[19] McDermid I, Barber M, Dennis M (2019). Home-Time Is a Feasible and Valid Stroke Outcome Measure in National Datasets. Stroke.

[20] Shen E, Rozema EJ, Haupt EC (2022). Assessing the concurrent validity of days alive and at home metric. J Am Geriatr Soc.

[21] Sung S-F, Su C-C, Hsieh C-Y (2020). Home-Time as a Surrogate Measure for Functional Outcome After Stroke: A Validation Study. Clin Epidemiol.

