# Supplementary material for: Measurement of quality of stroke care with national electronic health records: a prospective cohort study during and after the COVID-19 pandemic
Source: BMJ Open. 2026 May 19;16(5):e114881. doi: 10.1136/bmjopen-2025-114881 (PMC13202037; doi:10.1136/bmjopen-2025-114881)
Supplement: Supplementary file 3 [file bmjopen-2025-114881.reviewer_comments.pdf]

PEER REVIEW HISTORY

BMJ Open publishes all reviews undertaken for accepted manuscripts. Reviewers are asked to complete a checklist review form and are provided with free text boxes to elaborate on their assessment. These free text comments are reproduced below.

ARTICLE DETAILS

Title (Provisional)

Measurement of quality of stroke care with national electronic health records: a prospective cohort study during and after the COVID-19 pandemic

Authors

Farrell, James; Lambert, Roger; Nolan, John; Torralbo, Ana; Petersen, Steffen E.; Hocaoglu, Mevhibe; Tomlinson, Christopher; Sofat, Reecha; Huang, Qi; Kontopantelis, Evangelos; James, Martin; Lessels, Sarah; MacArthur, Jacqueline Ann Langdon; Wood, Angela; Whiteley, William; Denaxas, S; on behalf of the, CVD-COVID-UK/COVID-IMPACT Consortium

---

VERSION 1 - REVIEW

**Reviewer**	**1**
**Name**	**Ding, Qinglan**
**Affiliation**	**Purdue University, College of Health & Human Sciences**
**Date**	**28-Dec-2025**
**COI**	**None**

The study used linked-EHR data to assess stroke care quality in England before and after COVID-19 pandemic, focusing on stroke incidence, secondary prevention medications use, and a proxy for post stroke disability (time spent at home after stroke). The authors found that standardized annual stroke incidence increased from 227.6 to 244.8 per 100,000 over 2020-2023. Among ischemic stroke, reported first-year medication rates: 89.1% antiplatelet/anticoagulant, 44.5% antihypertensive, 80.5% lipid-lowering therapy. Secondary prevention medication use declined with increasing age and comorbidity, with limited variation by ethnicity/region/pandemic period. Based on these findings, the authors conclude that secondary prevention medication remains “underused”, particularly among older patients and those with comorbidity; linked EHRs could support scalable quality evaluation. The paper has multiple strengths such as the scale of the population studied, linkage across multiple EHR sources. That said, I have several comments for the authors’ consideration:

1. Please be clear about what the medication metric represents and avoid overinterpretation. The paper alternates between describing medication “prescription rates” and “dispensed medications”, while the limitations section clarifies that the analysis based on NHS dispensing data rather than prescribing records. Please use term consistently in the paper and abstract. Importantly, dispensing data alone cannot capture clinical intent or appropriateness of therapy, including clinical intent, contraindications, intolerance, end-of-life goals or patient preferences. Thus, statement implying that certain medication are “under-prescribed “ or that care quality is suboptimal may be too strong.

2. Relatedly, the absence of medication dispensing does not necessarily indicate inappropriate care. Without information on eligibility, contraindications, or prior intolerance, it is difficult to distinguish underuse from clinically appropriate non-use-particularly among older adults and patients with higher comorbidity burden. If possible, the authors should clarify eligibility criteria for each class or provide a more cautious interpretation of non-dispensing for each medication class.

3. For the conclusions, please try to align with what actually analyzed, with clearer differentiation between descriptive findings and broader claims about quality of care. The manuscript reports outcomes such as 30-day mortality and home time, but the main “care quality” inferences rely largely on medication dispensing rates.

**Reviewer**	**2**
**Name**	**Dack, Kyle**
**Affiliation**	**University of Bristol, MRC Integrative Epidemiology Unit**
**Date**	**13-Jan-2026**
**COI**	**None**

Statistical review summary:

The statistical analysis follows best practices and the methodology is clearly reported. My only concern is non-statistical – the framing of the paper seems somewhat inconsistent. In parts, it is described as an exploration of data quality (such as in title "measurement of quality", and the aims), but in other parts it is reported more like a purely descriptive study of epidemiological trends. To ensure readers interpret the results accurately, I have suggested including more guidance on how the results link back to the question of data quality.

Abstract:

1. The abstract is clear, containing a good overview of the study, and I do not have suggestions regarding it.

Introduction:

2. In the first two sentences it is stated twice that EHR linkage could improve case ascertainment – I am unsure whether different meanings are intended, but it seems slightly repetitive and may be worth rephrasing.

3. The introduction is well written, with clear aims. However, there seems a slight misalignment compared to the abstract (particularly outcome and result sections). The reported aims are to evaluate:

-ascertainment by source – this is not described in the abstract results.

-impact of COVID-19 on stroke recording – in the abstract this is framed as a comparison of incidence rather than recording quality.

-whether routine data can support quality measurement across 3 domains – the abstract reports 2 domains which are prescriptions and home time, but these are framed descriptively rather than as reflective of data quality.

Similarly, in the results, the framing seems slightly different –the results are sometimes framed as descriptive patterns rather than in terms of data quality evaluation. I think it would be helpful to link the results more directly to the stated aims, so that readers can clearly understand how the findings support the study conclusions.

Methods:

4. A minor comment but the first subsection “study design and data sources” does not directly report the study design (e.g. prospective cohort using EHR).

5. Data linkage: From my own experience of linking elements within NHS Test & Trace, there were a lot of edge cases or technical challenges which made perfect linkage impossible (e.g. missing NHS numbers, different addresses, etc). Perhaps the datasets used by the authors were cleaner, but if there are further details on the linking methodology it could be useful to include as a supplementary note. Or perhaps a sentence stating that data cleaning was already applied upstream by data providers.

6. The statistical analysis is reasonable and is clearly reported. However, similar to the previous comment, it would help readers if the alignment between the analysis and aims was made more explicit. While the introduction and aims emphasized that this is a comparison of data quality, the statistical analysis section does not really touch on this element of the study, and some readers may be left unsure as to how the results should be interpreted (e.g. whether differences represent variation in the quality of secondary prevention, or differences in ascertainment/coding across data sources).

7. Standardisation: I suggest adding information about standardization – the source and method applied, because this is first reported in the results.

Results

8. Table 1. I suggest adding a footnote to explain that there are two different denominators used for percentages. For the first row, the denominator is the total cohort (e.g. the top-right

cell), and for the rest of the data the denominator is the upper row (e.g. total GDPPR strokes). This is clear in-text but readers may skim and be confused why the first two rows of the table have similar N yet very different %.

9. The results and tables are well presented, consistent, and easy to follow. My only suggestion is to guide readers more in terms of how certain elements should be interpreted, e.g. paragraph 5 indicates there is minimal difference in stroke date between records, and I imagine this should be interpreted positively in terms of data linkage/comparability.

10. Tables S4/5 – were the assumptions of Cox's regression tested, in particular the proportional hazards assumption? It may be that because the statistics extracted from the models are not dependent on the assumption, it was decided to be unnecessary to test (this would be reasonable in my opinion). But if so, this should be clarified in the statistical analysis as readers may query this.

Discussion:

11. The discussion is clear and aligns with the results. I have no suggestions.

VERSION 1 - AUTHOR RESPONSE

Reviewer: 1 Dr. Qinglan Ding, Purdue University

Comments to the Author:
The study used linked-EHR data to assess stroke care quality in England before and after COVID-19 pandemic, focusing on stroke incidence, secondary prevention medications use, and a proxy for post stroke disability (time spent at home after stroke). The authors found that standardized annual stroke incidence increased from 227.6 to 244.8 per 100,000 over 2020-2023. Among ischemic stroke, reported first-year medication rates: 89.1% antiplatelet/anticoagulant, 44.5% antihypertensive, 80.5% lipid-lowering therapy. Secondary prevention medication use declined with increasing age and comorbidity, with limited variation by ethnicity/region/pandemic period. Based on these findings, the authors conclude that secondary prevention medication remains "underused", particularly among older patients and those with comorbidity; linked EHRs could support scalable quality evaluation. The paper has multiple strengths such as the scale of the population studied, linkage across multiple EHR sources.

Thank you for reviewing our work and the helpful suggestions all of which we have responded to below and by changing the manuscript.

That said, I have several comments for the authors' consideration: 1. Please be clear about what the medication metric represents and avoid overinterpretation. The paper alternates between describing medication "prescription rates" and "dispensed medications", while the limitations section clarifies that the analysis based on NHS

dispensing data rather than prescribing records. Please use term consistently in the paper and abstract.

Thank you for pointing out this inconsistency.

We have revised the abstract and main body of the manuscript to clarify that the study makes use of dispensed medications rather than prescriptions.

Importantly, dispensing data alone cannot capture clinical intent or appropriateness of therapy, including clinical intent, contraindications, intolerance, end-of-life goals or patient preferences. Thus, statement implying that certain medication are “under-prescribed “ or that care quality is suboptimal may be too strong. 2. Relatedly, the absence of medication dispensing does not necessarily indicate inappropriate care. Without information on eligibility, contraindications, or prior intolerance, it is difficult to distinguish underuse from clinically appropriate non-use-particularly among older adults and patients with higher comorbidity burden. If possible, the authors should clarify eligibility criteria for each class or provide a more cautious interpretation of non-dispensing for each medication class.

We agree that dispensing data does not capture the full clinical context, including contraindications, intolerance, or patient preferences etc. We have revised the manuscript to ensure our conclusions are more circumspect.

We have replaced 'under-prescribed' with terms like 'lower rates of dispensing' or 'potential gaps in therapy' throughout the Discussion.

We have added the following in the Discussion section:

> *While these findings suggest a potential gap in secondary prevention, the use of dispensing data alone cannot account for clinical contraindications, patient preferences, or end-of-life care goals that may appropriately preclude certain medications from being prescribed. Consequently, the observed lower rates of dispensing may reflect, in part, appropriate clinical decision-making that is not captured in routine EHR records.*

We have added the following in the Limitations section:

> *A further limitation is that our data lacks granular information on clinical intent, medication intolerance, and contraindications. We cannot distinguish between a failure to prescribe and a deliberate clinical decision based on patient frailty or preferences.*

We have additionally added a bullet point specifically to the point the reviewer raises in the "Strengths and limitations of this study" section:

> *The analysis is unable to account for medications prescribed in the private sector or clinical factors not captured in routine electronic health records, such as specific reasons for contraindications, prior tolerance, or eligibility to secondary prevention.*

3. For the conclusions, please try to align with what actually analyzed, with clearer differentiation between descriptive findings and broader claims about quality of care. The manuscript reports outcomes such as 30-day mortality and home time, but the main "care quality" inferences rely largely on medication dispensing rates.

Thank you for the suggestion.

We have revised the Conclusion section of the abstract accordingly:

> *Standardised stroke incidence increased significantly over the study period, highlighting a growing public health burden that persisted despite disruptions due to the pandemic. While secondary prevention coverage for antiplatelets and lipids was high, the suboptimal dispensing of antihypertensives, particularly in older and comorbid populations, signals a critical target for clinical optimisation. Home time represents a sensitive, person-centered outcome that exposes disparities linked to socioeconomic deprivation and clinical severity that can be used to enhance routine stroke audits. These findings justify the expansion of linked EHR infrastructure and the modernisation of governance frameworks to enable the longitudinal evaluation of care quality beyond the COVID-19 era.*

<u>Reviewer: 2 Dr. Kyle Dack, University of Bristol</u>

Comments to the Author: Statistical review summary: The statistical analysis follows best practices and the methodology is clearly reported. My only concern is non-statistical – the framing of the paper seems somewhat inconsistent. In parts, it is described as an exploration of data quality (such as in title "measurement of quality", and the aims), but in other parts it is reported more like a purely descriptive study of epidemiological trends. To ensure readers interpret the results accurately, I have suggested including more guidance on how the results link back to the question of data quality.

Thank you for reviewing our work and the helpful suggestions all of which we have responded to below and by changing the manuscript.

Abstract: 1. The abstract is clear, containing a good overview of the study, and I do not have suggestions regarding it.

Thank you.

Introduction: 2. In the first two sentences it is stated twice that EHR linkage could improve case ascertainment – I am unsure whether different meanings are intended, but it seems slightly repetitive and may be worth rephrasing.

We agree that the sentences are slightly confusing and have reworded these:

Near-real-time linkage of routine health and prescription records can enhance national stroke audits by improving case ascertainment and reducing the time and cost of data collection. Furthermore, integrating this data allows for the identification of care inequalities and the development of new quality measures, such as six-month disability indicators and secondary prevention coverage.

3. The introduction is well written, with clear aims. However, there seems a slight misalignment compared to the abstract (particularly outcome and result sections). The reported aims are to evaluate: -ascertainment by source – this is not described in the abstract results.
-impact of COVID-19 on stroke recording – in the abstract this is framed as a comparison of incidence rather than recording quality. -whether routine data can support quality measurement across 3 domains – the abstract reports 2 domains which are prescriptions and home time, but these are framed descriptively rather than as reflective of data quality. Similarly, in the results, the framing seems slightly different –the results are sometimes framed as descriptive patterns rather than in terms of data quality evaluation. I think it would be helpful to link the results more directly to the stated aims, so that readers can clearly understand how the findings support the study conclusions.

Thank you for pointing out this inconsistency in the framing of the results. We have revised the Abstract and Discussion parts of the manuscript to address this.

Methods: 4. A minor comment but the first subsection “study design and data sources” does not directly report the study design (e.g. prospective cohort using EHR).

We have revised the first sentence of the Methods section to explicitly state the study design:

We implemented a prospective cohort study and identified people over 18 years of age with a diagnosis of stroke between 1st January 2020 and 31st December 2023 and who were registered with a general practitioner (GP), living in England, had at least one record in General Practice Extraction Service Extract for Pandemic Planning and Research (GDPPR) and had a valid pseudo-identifier.

5. Data linkage: From my own experience of linking elements within NHS Test & Trace, there were a lot of edge cases or technical challenges which made perfect linkage impossible (e.g. missing NHS numbers, different addresses, etc). Perhaps the datasets used by the authors were cleaner, but if there are further details on the linking methodology it could be useful to include as a supplementary note. Or perhaps a sentence stating that data cleaning was already applied upstream by data providers.

Thank you for raising this point.

Individual data sources are linked by NHS Digital using the Master Person Service in combination with the Personal Demographics Service. A linkage score is calculated by cross-referencing information across different sources with the demographics in the Personal Demographics Service and signifies the overall associated match confidence. This score is not directly made available to researchers but NHS Digital's monthly reports for data quality maturity index indicate that 97-100% of records submitted to NHS Digital each month include information on NHS number and other key personal variables, providing confidence in the accuracy of the matching process - information is available here: https://digital.nhs.uk/data-and-information/data-tools-and-services/data-services/data-quality

We have revised the Methods section of the manuscript to further clarify:

> *Individual data sources were linked by NHS Digital using the Master Person Service in combination with the Personal Demographics Service and stringent data quality controls were applied upstream by NHS Digital to ensure data linkages were accurate.*

6. The statistical analysis is reasonable and is clearly reported. However, similar to the previous comment, it would help readers if the alignment between the analysis and aims was made more explicit. While the introduction and aims emphasized that this is a comparison of data quality, the statistical analysis section does not really touch on this element of the study, and some readers may be left unsure as to how the results should be interpreted (e.g. whether differences represent variation in the quality of secondary prevention, or differences in ascertainment/coding across data sources).

7. Standardisation: I suggest adding information about standardization – the source and method applied, because this is first reported in the results.

Thank you for pointing this out. We have included the following statement in the "Statistical analyses" section of the manuscript.

> *Monthly stroke incidence rates between 2020 and 2023 where age and sex standardised to the European Standard Population (ESP) using the direct method.*

Results 8. Table 1. I suggest adding a footnote to explain that there are two different denominators used for percentages. For the first row, the denominator is the total cohort (e.g. the top-right cell), and for the rest of the data the denominator is the upper row (e.g. total GDPPR strokes). This is clear in-text but readers may skim and be confused why the first two rows of the table have similar N yet very different %.

Thank you for the helpful suggestion - we have revised the legend for Table 1 to clarify this point:

> *Table 1 - Patient characteristics, demographics and stroke-related data for individuals with a first stroke between 2020 and 2023, stratified by the presence of a stroke record in the General Practice Extraction Service Data for Pandemic Preparedness (GDPPR), Hospital Episode Statistics Admitted Patient Care (HES-*

APC), Sentinel Stroke National Audit Programme (SSNAP), or Office for National Statistics (ONS) Deaths. The denominator for the top row of the table is the overall number of stroke events across all sources (n= 425,675) and for the rest of the rows the denominator is the total number of strokes recorded in each data source (e.g. 304,210 in GDPPR, 279,825 in HES-APC, 220,470 in SSNAP, and 59,465 in ONS).

9. The results and tables are well presented, consistent, and easy to follow. My only suggestion is to guide readers more in terms of how certain elements should be interpreted, e.g. paragraph 5 indicates there is minimal difference in stroke date between records, and I imagine this should be interpreted positively in terms of data linkage/comparability.

Thank you for pointing this out, we have revised the discussion to address this comment and have additionally added the following statement:

While each individual data source captured stroke events that were uniquely recorded in that source, the overall concordance of stroke events between sources was very high (median 0 days between records) indicating both a good level of data linkage across sources and quality within sources.

10. Tables S4/5 – were the assumptions of Cox's regression tested, in particular the proportional hazards assumption? It may be that because the statistics extracted from the models are not dependent on the assumption, it was decided to be unnecessary to test (this would be reasonable in my opinion). But if so, this should be clarified in the statistical analysis as readers may query this.

Thank you for raising this point. The proportional hazard assumption for Cox models was not formally evaluated, as the estimated hazard ratios were intended to be interpreted as time-average effects. We have now clarified this in the Methods by adding the following:

Formal evaluation of the proportional hazards assumption was not performed, as the estimated hazard ratios were intended to summarise time-averaged effects over the follow-up period rather than to explore time-varying associations.

Discussion: 11. The discussion is clear and aligns with the results. I have no suggestions.

Thank you.

VERSION 2 - REVIEW

**Reviewer**	**1**
**Name**	**Ding, Qinglan**
**Affiliation**	**Purdue University, College of Health & Human Sciences**

Date 10-Apr-2026

COI

The revision addresses many prior concerns, a few minor issues worth the authors attention:

1. The revision appropriately acknowledges that dispensing data do not capture clinical intent/eligibility, contraindications, intolerance, frailty/end of life goals, or patient preference. However, in the revision's many places, the terms such as "underuse, suboptimal" still exist. These language might risk over interpretation and should be replaced by neutral descriptors.

2. In the methods of the paper, the revision introduces definitions that imply an incident medication window beginning one month after discharge. But in the abstract and headline result refers to first year dispensing rates without clearly stating whether the first month is excluded. Clarification of this 1 month washout can help enhance interpretation.

3. In the abstract conclusion, it currently reads as if the observed increase in age/sex-standardized incidence represents a true population increase and therefore a growing public health burden. But the paper itself also recognizes variation in ascertainment across sources and potential coding limitations. The incidence statement or conclusion of the paper should be reframe more carefully.

Reviewer 2

Name Dack, Kyle

Affiliation University of Bristol, MRC Integrative Epidemiology Unit

Date 23-Mar-2026

COI

No further comments - it is an interesting and nicely written paper.

VERSION 2 - AUTHOR RESPONSE

Reviewer: 1 Dr. Qinglan Ding, Purdue University

Comments to the Author:
The revision addresses many prior concerns, a few minor issues worth the authors attention:
1. The revision appropriately acknowledges that dispensing data do not capture clinical intent/eligibility, contraindications, intolerance, frailty/end of life goals, or patient preference. However, in the revision's many places, the terms such as "underuse, suboptimal" still exist. These language might risk over interpretation and should be replaced by neutral descriptors.

We have revised the manuscript and replaced offending terms.

2. In the methods of the paper, the revision introduces definitions that imply an incident medication window beginning one month after discharge. But in the abstract and headline result refers to first year dispensing rates without clearly stating whether the first month is excluded. Clarification of this 1 month washout can help enhance interpretation.

We have revised the abstract to specify the washout period:

> *first-year medication dispensing rates for antiplatelets, anticoagulants, antihypertensives, and lipid-lowering agents (with a one-month washout period)*

3. In the abstract conclusion, it currently reads as if the observed increase in age/sex-standardized incidence represents a true population increase and therefore a growing public health burden. But the paper itself also recognizes variation in ascertainment across sources and potential coding limitations. The incidence statement or conclusion of the paper should be reframe more carefully.

We have revised the abstract Conclusion to further clarify:

> Standardised stroke incidence increased significantly over the study period, highlighting a growing public health burden that persisted despite disruptions due to the pandemic although it should be noted that variation in case ascertainment and stroke coding practices was observed.
